# Methane transport in agricultural soil after injection of isotopically-enriched methane in the sub-surface

**DOI:** 10.1038/sdata.2018.208

**Published:** 2018-10-16

**Authors:** George Shaw, Brian Atkinson, William Meredith, Colin Snape, David Lever, Andrew Hoch

**Affiliations:** 1 School of Biosciences, University of Nottingham, Nottingham, United Kingdom; 2 School of Engineering, University of Nottingham, Nottingham, United Kingdom; 3 Wood plc, Harwell, United Kingdom

**Keywords:** Environmental chemistry, Carbon cycle

## Abstract

Small quantities of radioactive methane (^14^CH_4_) may be released over prolonged periods from geological disposal facilities for radioactive waste. The impact of this release depends on the capacity of soil to oxidise ^14^CH_4_ to ^14^CO_2_ during transport from the sub-surface to the atmosphere. We investigated this capacity by pulse-injecting isotopically-enriched methane 50 cm below the surface of an agricultural soil in central England. Three sequential injections were made during growth of a spring wheat crop. Samples of gas were taken from the pore space throughout the soil profile at predetermined time points after injection, accompanied by samples of the atmosphere above the soil collected in sampling chambers, deployed at scheduled intervals. Methane and CO_2_ were measured in soil and above-ground gas using gas chromatography; the isotopic composition of CH_4_ and CO_2_ was determined using gas chromatography with isotopic ratio mass spectrometry. Supporting measurements of environmental variables were made during the experiment. The data can be used to test mathematical models describing CH_4_ and CO_2_ transport and fate in temperate agricultural soils.

## Background & Summary

This study addresses the capacity of a typical agricultural soil to transport and oxidise methane arising in the shallow sub-soil as it diffuses towards the free atmosphere above the soil surface. The study was carried out in the context of radioactive waste disposal in a geological disposal facility (GDF) which could give rise to radiation doses to humans and other organisms in the environment above the repository^1^. Radioactive gases, including ^14^CH_4_, can be produced in a geological repository due to corrosion of metals and decomposition of organic materials^2^. Transport of ^14^CH_4_ from a GDF through the geosphere can occur in the dissolved phase, possibly in association with ebullition and transport as small gas bubbles^3^. Having reached the sub-soil, the degree of transport of this radioactive methane to the soil surface is dependent on diffusion through the soil and the potential for oxidation to ^14^CO_2_ by the methanotrophic microflora during transport. Calculations of the radiation doses potentially received from exposure to ^14^C via this pathway depend on assumptions of the degree of conversion of ^14^CH_4_ to ^14^CO_2_ during migration through the soil profile. Significant conversion to ^14^CO_2_ is likely to lead to greater exposure since, in this form, ^14^C can be photosynthetically fixed by crop canopies as the gas diffuses from the soil surface into the atmosphere within the plant canopy boundary layer.

**Figure 1:**
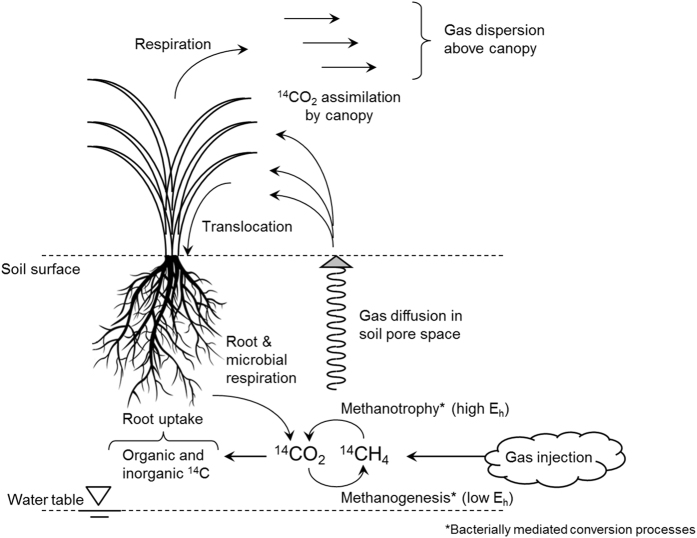
Conceptual model of mechanisms governing the fate of ^14^CH_4_ and ^14^CO_2_ following the introduction of ^14^CH_4_ into the sub-soil.

The specific objective of this study was to obtain experimental data on the behaviour of CH_4_ and CO_2_ in the soil zone, when CH_4_ arises from depth. The key processes we sought to capture in the study are summarised schematically in Fig. 1. To ensure that the data obtained are as relevant as possible to ambient environmental conditions a field experiment was designed and implemented. This involved the injection of discrete pulses of methane at a depth of 50 cm below the surface of a typical, productive agricultural soil in which a full-scale spring wheat crop was cultivated. The methane injected into the sub-soil was highly isotopically enriched to ensure it could be unequivocally distinguished from ambient methane and to facilitate the identification of CO_2_ formed as a result of oxidation of the injected methane. Time-course samples were taken to determine the fate of the injected methane in the soil gas and the atmosphere above the soil surface. Using these samples, total (‘bulk’) methane concentrations and the isotopic signature (δ^13^C) of methane were measured above the points where the methane had been injected. The same samples were used to measure total (‘bulk’) CO_2_ concentrations and the isotopic signature (δ^13^C) of CO_2_ to determine the degree of oxidation, if any, of the injected methane to CO_2_. The field experiment (and supporting laboratory experiments) has previously been reported by Atkinson *et al.*^4^. As part of the study, appropriate models at different levels of detail were developed to facilitate a full interpretation of experimental results within the context of the specific aims of the experiment^5,6^.

Our study was focused on the requirement to assess the potential for radiation doses arising from exposure to ^14^C, originating as ^14^CH_4_ in the sub-surface beneath an arable ecosystem. The behaviour and fate of CH_4_ and CO_2_ in soils are of much wider significance, however, in the context of sink-source relationships between greenhouse gases in the atmosphere and the land surface, and carbon storage in the terrestrial environment. The data from our study are, therefore, applicable in a much wider context, including testing mathematical models describing methane and CO_2_ transport and fate in agricultural soils under temperate climates. Our data set also incorporates supporting information, including high resolution data on soil moisture, soil temperature and meteorology, which is applicable in testing models which require these physical parameters as key inputs to their calculations or which aim to make predictions of these physical variables under field conditions.

## Methods

### Location and timing

The field experiment was carried out at Bunny Park, Nottinghamshire, UK (52.863°N, 1.126°W, 39 m above mean sea level). The soil at the experimental site is a stagno-gleyic brown earth (loamy sand texture) of the Newport series. Gravel and cobbles are prevalent below a depth of 30–40 cm and the site overlies a solid geology of Triassic mudstone. The experimental methane injections were made in June and July 2012, with final sampling and disassembly of the experiment in August 2012.

### Cultivation

**Figure 2:**
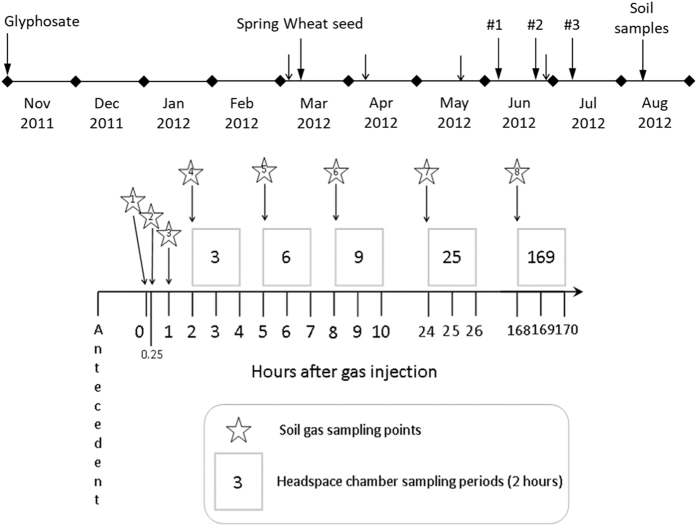
Timeline of the field experiment (above). Larger vertical arrows indicate major events; smaller arrows indicate applications of fertiliser, herbicide and fungicide as described under ‘Cultivation’ in the Methods section. The three time points at which aliquots of isotopically-enriched methane were injected 50 cm below the soil surface of the experimental plots are indicated by #1, #2 and #3. The detailed schedule (below) shows the timings of soil gas and ‘headspace’ chamber sampling within each experimental plot following each injection of CH_4_. Zero on the horizontal axis represents the time point at which CH_4_ was injected. Headspace chambers were placed on the soil surface above the injection points to measure the emergence of CH_4_ and CO_2_ from the soil into the free atmosphere. The timing of headspace chamber placements was carefully arranged to avoid interference with soil gas sampling.

An area of approximately 11,000 m^2^ (1.1 Ha) was left fallow following removal of a crop of ryegrass, using Glyphosate, on 1^st^ November 2011. Cultivation was carried out in early March 2012 by ploughing and harrowing. On 12^th^ March the area was seeded with spring wheat (*Triticum aestivum* cv. Tybalt – Redigo) at a density of 300 seeds per m^2^. Applications of herbicide were made on 23^rd^ May 2012, and fungicide was applied on 23rd May and 25^th^ June 2012. Ammonium nitrate was applied on 8^th^ March and 11^th^ April 2012, and manganese on 29th June 2012. The field surrounding the experimental area was planted with a guard crop of oil seed rape (*Brassica napus*) in autumn 2011. The overall time-line of the experiment is shown in the upper part of Fig. 2.

### Plot design

**Figure 3:**
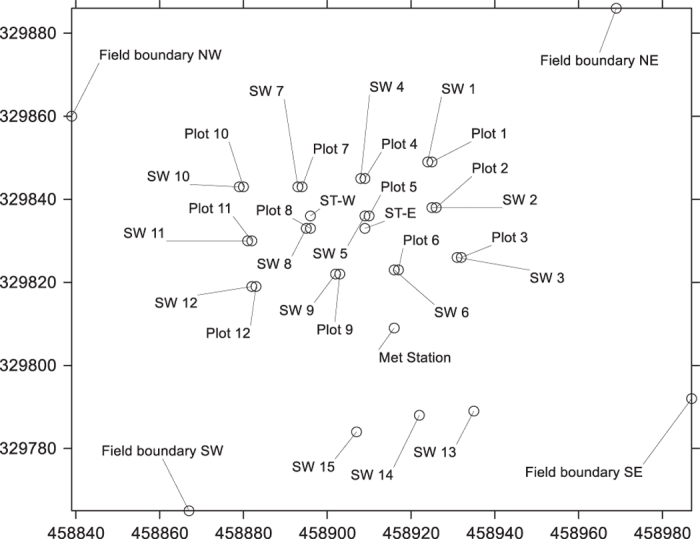
Map showing individual plot locations (numbered Plot 1 to Plot 12), individual soil moisture monitoring points (numbered SW 1 to SW 15), soil temperature monitoring points (ST-W and ST-E) and the position of the automatic meteorological station (Met Station). Axes are northings and eastings on the Ordnance Survey grid of Great Britain (units of distance are metres).

Twelve experimental plots were established in a 3 × 4 grid covering an area of 45 m × 25 m in the centre of the area planted with spring wheat. Six randomly-selected plots were maintained with continuous wheat cover. The remaining six ‘no-plant’ plots were created by removing wheat plants down to ground level in a circular area of 1.5 m diameter around the centre of each plot. The layout of the twelve plots and associated instrumentation is shown in Fig. 3. Plots 2, 3, 7, 9, 11 and 12 were ‘no-plant’ treatments. Individual geographical coordinates of all experimental plots can be found in Field_Locations_2012.csv (Data Citation 1).

### Plot installation and instrumentation

In each experimental plot, five gas sampling tubes of varying length, as described by Huxtable *et al.*^7^, were inserted at a 45° angle to the soil surface. These were arranged so that their perforated sampling tips were positioned in the vertical dimension at 10, 20, 30, 40 and 50 cm depths from the soil surface below the centre of each experimental plot. A circular PVC collar (50 cm diameter, 10 cm depth) was inserted into the soil to a depth of 5 cm around the centre of each plot to provide a gastight seat for sampling chambers.

Vertical access tubes for in situ soil moisture measurements were inserted to a depth of 45 cm at 15 monitoring points, as indicated in Fig. 3. Twelve of these soil moisture monitoring points (SW1 to SW12 in Fig. 3) were located adjacent to each experimental plot, 1.25 m from the centre of each plot. The remaining three (SW13 to SW15 in Fig. 3) were located approximately 35 m to the south of the experimental plots. Soil moisture was measured in these access tubes at depths of 10, 20, 30 and 40 cm using a Delta-T PR2 profile probe and an HH2 moisture meter (Delta-T Devices, Cambridge, UK). In situ soil moisture measurements were taken manually each week at all 15 monitoring points. Data from these measurements can be found in Soil_Moisture_2012.csv (Data Citation 1). At the end of the experiment, soil pits were excavated as close as possible to each in situ soil moisture monitoring point. From these pits, intact soil samples of fixed volumes were taken at 5 cm intervals from the soil surface to a depth of 50 cm. These were used to measure gravimetric water content, saturated water content (used as a measure of soil porosity) and dry bulk density. Data from these measurements can be found in Soil_Porosity_&_Bulk_Density_2012.csv (Data Citation 1).

Two vertical arrays of thermocouple cables (supplied by TC Ltd., http://www.tc.co.uk) were installed at depths of 10, 20, 30 and 40 cm, located close to the centre of the 45 m × 25 m experimental area (ST-W and ST-E in Fig. 3). Soil temperatures were recorded automatically throughout the period of the experiment at 15 min intervals using a Campbell 21X data logger. Data from these measurements can be found in Soil_Temperature_2012.csv (Data Citation 1).

A full suite of meteorological data was collected from 8^th^ May to 13^th^ August 2012. Rainfall, air temperature, humidity, barometric pressure, wind speed and direction were recorded automatically over 30 min intervals using a Davis Vantage Pro 2 wireless weather station located approximately 30 m south of the centre of the experimental area. Data from these measurements can be found in Meteorological_Data_2012.csv (Data Citation 1).

Individual geographical coordinates of all monitoring points (soil moisture, soil temperature and meteorological) can be found in Field_Locations_2012.csv (Data Citation 1).

### Preparation, injection and sampling of gases

Isotopically-pure sources of ^12^CH_4_ (99.95% ^12^C) and ^13^CH_4_ (99.0% ^13^C) were obtained from CK Gases, Hook, UK. In the laboratory, small volumes of each gas were dispensed using a Hamilton® 10 ml gastight syringe and mixed at a ratio of 7:3 (^12^CH_4_ : ^13^CH_4_) using a bubble trap apparatus; this allowed accurate volumes of each gas to be obtained at atmospheric pressure while eliminating any loss of gas during the dispensing procedure. The resulting δ^13^C value (with respect to PD belemnite) was 37,139‰. After preparing the mixed ^12/13^CH_4_ in the laboratory, 15 ml aliquots were dispensed at ambient temperature and pressure into pre-evacuated glass Exetainer® vials (supplied by Labco, Lampeter, UK), fitted with rubber septa, prior to injection of the gas in the field experiment.

Eight randomly selected plots (plot numbers 1, 2, 4, 5, 7, 10, 11 and 12) received CH_4_ injections. Four control plots (plot numbers 3, 6, 8 and 9 - two with and two without wheat) did not receive CH_4_ injections.

In the field, aliquots of isotopically-enriched CH_4_ were extracted from Exetainer® vials using a Hamilton® 10 ml gastight syringe and injected into the sub-soil (50 cm from the soil surface) through the deepest gas sampler in each ‘gassed’ plot. This was performed in two pulse injection volumes of 7.5 ml, giving a total injected volume of 15 ml. A small pulse of ambient air (2 ml) was then injected into the 50 cm sampler to push any residual CH_4_ from the sampler tube and into the soil; the 50 cm sampler was then closed using a stopcock to eliminate any leakage of CH_4_ back through the sampler. Gas injections were performed on three separate occasions for each ‘gassed’ plot over a seven week period. Due to the need to sample above-ground and soil gases shortly after injecting the CH_4_, two experimental plots were injected with CH_4_ per day over four consecutive days. The first gas injections took place between the 11th and 14th June 2012, the second injections took place from 25th to 28th June 2012, and the third and final injections were performed between the 16th and 19^th^ July 2012. Antecedent soil gas samples were taken from all plots 2–3 weeks prior to the first gas injection round.

Samples of gas from the pore space of the soil were collected using the previously installed soil gas sampling tubes. Soil gas samples at all depths were collected using a 20 ml gastight syringe immediately prior to CH_4_ injection and at eight pre-determined time-points following each gas injection (0.25, 1, 2, 5, 8, 10, 24, and 168 h) covering a period from 15 min to 7 days. Soil gas samples were injected into pre-evacuated Exetainer® vials (14 ml volume) and taken to the laboratory for analysis of concentrations and isotopic composition of CH_4_ and CO_2_ (described below).

A clear PVC sampling chamber 50 cm in diameter and either 50 or 100 cm in height (depending on the growth stage of the crop) was used to collect gases emerging from the soil surface into the atmosphere above. This ‘headspace’ chamber was located snugly on the circular PVC collar (previously installed above each of the experimental plots) at intervals from 3 h to 168 h after injecting methane pulses 50 cm below the soil surface. These sampling intervals were carefully timed to avoid interference with soil gas sampling (the schedule of CH_4_ injection and gas sampling is shown schematically in the lower part of Fig. 2). After placing the chambers over each plot, samples of gas within each chamber were taken with a 20 ml gastight syringe at 30 min intervals over a 120 min period during which the atmosphere within the chamber was continuously stirred with a small electric fan. After withdrawal from the chambers, gas samples were injected into pre-evacuated Exetainer® vials (14 ml volume). After 120 min the chambers were removed from the plots and the gas samples taken to the laboratory for analysis of concentrations and isotopic composition of CH_4_ and CO_2_ (described below).

### Analysis of gas samples

Bulk concentrations of CH_4_ and CO_2_ in gas samples were determined using a gas chromatograph (GC-2014, Shimadzu Corp., Japan) fitted with thermal conductivity and flame ionisation detectors (FID) in parallel. Aliquots (5 ml) of gas samples from the soil gas and headspace chambers were withdrawn by Hamilton® gastight syringe from the Exetainer® vials and injected into the GC-FID via an injection loop. Measurements of peak area were then compared with calibrated gas standards (supplied by SIP Analytical, Sandwich, Kent) containing 493 ppmv CO_2_ and 53.3 ppmv CH_4_. Carbon dioxide and CH_4_ concentrations were then calculated based on the average peak area of the appropriate standard and the peak area of the sample. Bulk CH_4_ and CO_2_ concentrations in all gas samples are expressed in parts per million by volume (ppmv).

Bulk concentration data resulting from soil gas measurements can be found in:

Methane_in_Soil_2012.csv (Data Citation 1), and

Carbon_Dioxide_in_Soil_2012.csv (Data Citation 1).

Bulk concentration data resulting from headspace gas measurements can be found in:

Methane_in_Headspace_2012.csv (Data Citation 1), and

Carbon_Dioxide_in_Headspace_2012.csv (Data Citation 1).

The ^13^C/^12^C isotope ratios for CO_2_ and CH_4_ were determined using a DeltaplusXP, ThermoFinnigan gas chromatography-combustion-isotope ratio mass spectrometer (GC-C-IRMS). Injection of samples was performed in split mode (ratio 3:1, injection temperature 60 °C), with separation performed on a Varian CP-PoraPLOT Q-HT column (10 m × 0.53 mm; 20 μm film thickness) with helium as the carrier gas (1.5 ml min^−1^), at a temperature of 30 °C held isothermally for 20 min. Each 20 min run included two initial pulses of standard CO_2_, introduced directly in the mass spectrometer (with all data calculated against pulse 2), followed by an injection of the same standard CO_2_ (5 μl) to assess any fractionation during passage through the GC and combustion furnace. Then followed three replicate injections of the gas sample under test (amounts varied according to CO_2_ concentration, but typically these were 1 ml in volume). A second injection of the standard CO_2_ (5 μl) was made to check for fractionation over the course of the run, followed by three pulses of the standard CO_2_, again introduced directly into the mass spectrometer, to assess instrument accuracy.

Each sample injection resulted in three well-resolved chromatographic peaks. The first is a peak dominated by m/z 46 (m/z = ratio of mass to charge for individual peaks identified in the chromatogram) which is due to NO_2_ derived from oxidation of nitrogen within the sample. The second peak is CO_2_ derived from the combustion of methane, and this is followed by the peak of CO_2_ from the sample which passes through the combustion furnace unaltered. The ^13^C/^12^C isotopic ratio of the CH_4_ and CO_2_ in each sample was calculated as the average value from the three replicate analyses. Isotopic composition of CH_4_ and CO_2_ in all gas samples is expressed as a δ^13^C value normalised to PD belemnite in units of ‰ (per mille).

Isotopic data resulting from soil gas measurements can be found in:

Delta-13C_Methane_in_Soil_2012.csv (Data Citation 1), and

Delta-13C_Carbon_Dioxide_in_Soil_2012.csv (Data Citation 1).

Isotopic data resulting from headspace gas measurements can be found in:

Delta-13C_Methane_in_Headspace_2012.csv (Data Citation 1), and

Delta-13C_Carbon_Dioxide_in_Headspace_2012.csv (Data Citation 1).

### Code availability

The main code we used to interpret the experimental data was TOUGH2. This is proprietary software; the IP is owned by Lawrence Berkeley National Laboratory. However, the version of the software that we used is available for free download by registered organisations from the NEA Data Bank, and is available at the web site https://www.oecd-nea.org/tools/abstract/detail/ests0219.

TOUGH2 is distributed with a set of ‘Equation Of State (EOS)’ modules. One of those modules is called EOS7 and is usually used to model water, water vapour, a bulk gas component (air) and two trace gas components. We adapted that module (to compute the density, viscosity, etc. of a different mixture of gases) to our problem, in particular making it possible to simulate water, water vapour, oxygen, nitrogen, carbon dioxide, methane, ^13^C-labelled carbon dioxide and ^13^C-labelled methane.

The terms of our licence with the NEA Data Bank prohibit us from distributing either TOUGH2 or any modifications to the code.

## Data Records

All data from the field experiment described are available from the NERC Environmental Information Data Centre (Data Citation 1). The data set consists of comma-separated text files (^∗^.csv), as listed in Table 1. Each data file is accompanied by a descriptor file in rich text (^∗^.rtf) format which lists the column headings in each data file and also provides a brief explanation of the data file contents, the units used for each parameter and, where applicable, further explanatory notes.

## Technical Validation

The primary potential sources of uncertainty in the data set lie in the preparation of isotopically-enriched CH_4_ mixtures and analysis of CH_4_ and CO_2_ in soil gas and above-ground (‘headspace’) gas samples.

Primary dispensing and sampling of gases were carried out using gastight glass Hamilton® syringes (Hamilton Company, USA) which were routinely checked for both accuracy and precision by gravimetric analysis using ultrapure (18 MΩ) water.

In the preparation of mixed isotopic sources of methane, isotopically-pure sources of ^12^CH_4_ (99.95% ^12^C) and ^13^CH_4_ (99.0% ^13^C) were obtained from CK Gases, Hook, UK. In subsequent measurements of all gas samples, high purity certified calibration gases for gas chromatography (GC) analyses of bulk concentrations of CH_4_ and CO_2_ were obtained from SIP Analytical (Sandwich, Kent). All GC analyses were carried out within a strict quality assurance protocol in which analyses for both CH_4_ and CO_2_ were consistently checked against the certified values for the calibration gases. During the course of analysis of samples from the field experiment, 88% of GC analyses were within a 3% tolerance envelope, the remaining 12% within a 5% tolerance envelope, based on the certified concentrations of the calibration gases.

Analyses of the isotopic composition of CH_4_ and CO_2_ in gas samples were subject to a rigorous protocol, as described in the methods section, above. The standard used during the IRMS analysis was 99.999% pure CO_2_ obtained from Air Products PLC (Walton-on-Thames, Surrey, UK). The original δ^13^C value of the CO_2_ standard was calibrated by Iso-Analytical (Crewe, UK) using 10 individual sub-samples of the gas. Further calibrations were made using a standard alkane mix (A6) obtained from the Arndt Schimmelmann laboratory in the Department of Earth and Atmospheric Sciences, Indiana University, USA.

Meteorological data collected at the site of the experiment were cross-checked against data available from the nearest weather station operated by the Meteorological Office (UK), which is located at Sutton Bonington, 8.8 km WSW of the Bunny experimental field plot (52.833°N 1.250°E, 48 m above mean sea level). For average daily air temperature the agreement between data from the two sites was almost perfect (R^2^ = 0.99). For daily rainfall the R^2^ value was 0.83, reflecting the more localised nature of rainfall compared with air temperature. Data available from the Meteorological Office weather station also include soil temperatures, which were used to cross-check soil temperature data collected by a data logger at the site of the experiment. Comparison of soil temperatures at 10 and 30 cm depths from our field site and the Meteorological Office weather station produced R^2^ values of 0.87 and 0.82, respectively. In situ measurements of soil moisture using the Delta-T PR2 profile probe and an HH2 moisture meter were validated against gravimetric measurements of soil moisture taken by destructive sampling of soil pits at the end of the field experiment.

The everyday project management was carried out under quality assurance systems which fully meet the requirements of ISO 9001:2008. The field experiment (and related supporting laboratory experiments) was subject to an external quality assurance review on 9^th^ August 2012. This was conducted by Radioactive Waste Management (UK). This review covered, inter alia, traceability of data records from field sampling and laboratory analysis to the final deposition of data in an in-house data base. The review identified evidence of good practice, including methodical calibration processes (as described above), traceability of raw data to calibration records and clear in-house operating procedures.

## Usage Notes

Users of the data will find it informative, as a starting point, to refer to the data analyses described in the reports by Hoch *et al*.^5,6^ which provide an analysis of the findings of the study and detailed modelling interpretations of the data. The findings of these analyses are summarised below, which is an edited summary from Hoch *et al.*^5^.

**Figure 4:**
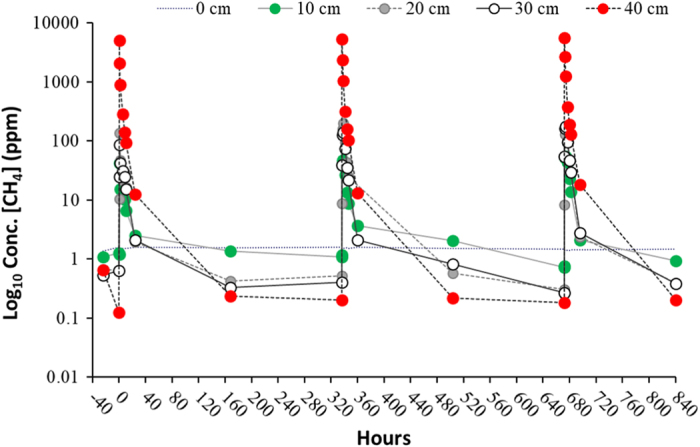
Bulk methane concentrations in soil gas in response to three consecutive sub-surface injections of ^12/13^CH_4_ in a field experiment with spring wheat. Zero on the X axis represents the time at which ^12/13^CH_4_ was injected. Methane concentrations at −24 and 0 h are ‘antecedent’ measurements.

**Figure 5:**
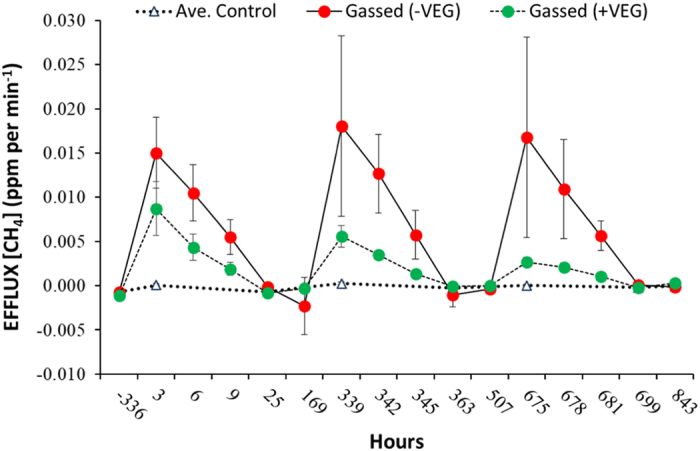
Efflux of bulk methane from the soil surface to the atmosphere above the soil in response to three consecutive sub-surface injections of ^12/13^CH_4_ in a field experiment with spring wheat. Points are arithmetic means (n = 4), vertical bars are SEM (NB. The scale on the X-axis is non-linear).

**Figure 6:**
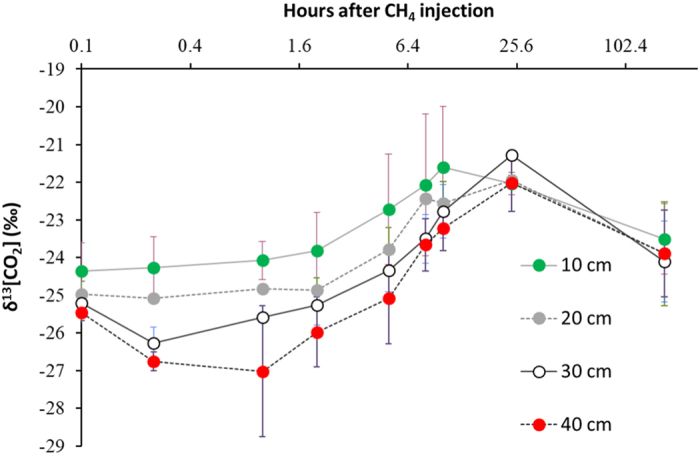
δ^13^CO_2_ in soil gas samples in response to in response to a sub-surface injection of ^12/13^CH_4_ in a field experiment with spring wheat. Points are arithmetic means (n = 2), vertical bars are SEM.

Following three injections of methane (^12^CH_4_ and ^13^CH_4_) 50 cm below the soil surface, consistent measurements have been made of the profiles of gas concentrations (^12^CH_4_, ^13^CH_4_, ^12^CO_2_ and ^13^CO_2_) throughout the soil profile and efflux of gases from the soil surface (Figs 4 and 5). As methane diffuses away from the point of injection, it is oxidised to carbon dioxide by the methanotrophic microflora as evidenced by the shift in δ^13^CO_2_ in soil gas samples (Fig. 6). The carbon dioxide (^12^CO_2_ and ^13^CO_2_) then diffuses upwards through the partially saturated soil and into the overlying atmosphere. Small volumes of methane were injected so as not to stimulate the activity of methanotrophic populations. As a result, the uptake of labelled carbon dioxide by the spring wheat crop was below detection limit. However, the experiment allowed three key processes in the soils to be examined:

- diffusion of gases through partially saturated soil;
- microbial oxidation of methane; and
- soil respiration (ie. microbial degradation of soil organic matter to produce carbon dioxide which enriches the background concentration profile of CO_2_ in the soil).

A computational model was developed that accounts for all of these processes, as well as isotopic effects (different isotopic forms of a gas are expected to have slightly different rates for each process). In applying the model to interpret the experimental data, we made use of previously published relationships describing the effective diffusion coefficient as a function of soil properties^8,9^. These model relationships provide estimates of the rates of gas diffusion through the soil with relatively low uncertainty.

The numerical model was able to replicate most of the gross features of the experiments, apart from more rapid consumption of ^13^CH_4_ than ^12^CH_4_. The combination of the experimental data and numerical modelling allowed us to determine the rate at which microbes convert methane, via an intermediate form, to carbon dioxide.

The numerical model was complemented by simpler models used to analyse the antecedent ‘headspace’ and soil profile measurements, assuming a soil profile with homogeneous properties and steady-state conditions. They were also used to estimate the proportion of ^14^CH_4_ that left the system. The characteristic length scale (m) over which methane will be oxidised in the soil to carbon dioxide is given by $\sqrt{D/(S_{g}k)}$ where D is the effective diffusion coefficient of methane in the soil (m^2^ s^−1^), *ϕ* is the soil porosity (dimensionless), S_g_ is the soil gas saturation (also dimensionless) and k is the first-order rate coefficient for oxidation of methane (s^−1^). Antecedent measurements of methane in the field provided values of k from 1.9 × 10^−5^ s^−1^ to 2.4 × 10^−4^ s^−1^, with corresponding characteristic length scales in the range 0.072 to 0.27 m. Methane oxidation rate coefficients following sub-surface injection of methane ranged from 4 × 10^−5^ s^−1^ to 6 × 10^−5^ s^−1^.

Although the characteristic length scale for methane oxidation will be specific to the site and ecosystem under consideration, it seems generally to be of the order of tens of centimetres in agricultural or arable environments. The implication is that most of the radioactive methane migrating from a deep radioactive waste repository is likely to be converted to radioactive carbon dioxide in the soil. There is then the potential for the uptake of radioactive carbon dioxide by plants if this efflux from the soil continues over prolonged periods.

## Additional information

**How to cite this article**: Shaw, G. *et al.*, Methane transport in agricultural soil after injection of isotopically-enriched methane in the sub-surface. *Sci. Data*. 5:180208 doi: 10.1038/sdata.2018.208 (2018).

**Publisher’s note**: Springer Nature remains neutral with regard to jurisdictional claims in published maps and institutional affiliations.

## Supplementary Material



## Figures and Tables

**Table 1:** Listing of files, in comma-separated (*.csv) format, containing data originating from the field study to determine methane transport through an agricultural soil following pulse injection of isotopically-enriched methane in the sub-surface.

**Sample**	**Geographical location**	**Geoposition**	**Protocol**	**Data**
Gas samples in headspace above experimental plots	Bunny Park, Nottinghamshire (UK)	52.863°N, 1.126°W39 m amsl	Measurement of bulk carbon dioxide concentrations in headspace gas samples	Carbon-Dioxide_in_Headspace_2012.csv
Gas samples from soil below experimental plots	Bunny Park, Nottinghamshire (UK)	52.863°N, 1.126°W39 m amsl	Measurement of bulk carbon dioxide concentrations in soil gas samples	Carbon-Dioxide_in_Soil_2012.csv
Gas samples in headspace above experimental plots	Bunny Park, Nottinghamshire (UK)	52.863°N, 1.126°W39 m amsl	Measurement of bulk methane concentrations in headspace gas samples	Methane_in_Headspace_2012.csv
Gas samples from soil below experimental plots	Bunny Park, Nottinghamshire (UK)	52.863°N, 1.126°W39 m amsl	Measurement of bulk methane concentrations in soil gas samples	Methane_in_Soil_2012.csv
Gas samples in headspace above experimental plots	Bunny Park, Nottinghamshire (UK)	52.863°N, 1.126°W39 m amsl	Measurement of isotopic composition of carbon dioxide in headspace gas samples	Delta-13C_Carbon_Dioxide_in_Headspace_2012.csv
Gas samples from soil below experimental plots	Bunny Park, Nottinghamshire (UK)	52.863°N, 1.126°W39 m amsl	Measurement of isotopic composition of carbon dioxide in soil gas samples	Delta-13C_Carbon_Dioxide_in_Soil_2012.csv
Gas samples in headspace above experimental plots	Bunny Park, Nottinghamshire (UK)	52.863°N, 1.126°W39 m amsl	Measurement of isotopic composition of methane in headspace gas samples	Delta-13C_Methane_in_Headspace_2012.csv
Gas samples from soil below experimental plots	Bunny Park, Nottinghamshire (UK)	52.863°N, 1.126°W39 m amsl	Measurement of isotopic composition of methane in soil gas samples	Delta-13C_Methane_in_Soil_2012.csv
Soil moisture	Bunny Park, Nottinghamshire (UK)	52.863°N, 1.126°W39 m amsl	Manual measurement of soil moisture content	Soil_Moisture_2012.csv
Soil porosity and bulk density	Bunny Park, Nottinghamshire (UK)	52.863°N, 1.126°W39 m amsl	Manual measurement of soil porosity and bulk density	Soil_Porosity_&_Bulk_Density_2012.csv
Soil temperature	Bunny Park, Nottinghamshire (UK)	52.863°N, 1.126°W39 m amsl	Automated measurement of soil temperature	Soil_Temperature_2012.csv.
Meteorological variables	Bunny Park, Nottinghamshire (UK)	52.863°N, 1.126°W39 m amsl	Automated measurement of meteorological variables	Meteorological_Data_2012.csv
